# Stress and Gastroesophageal Reflux Disease: Are They Related?

**DOI:** 10.7759/cureus.79037

**Published:** 2025-02-15

**Authors:** Muhammad Sajeel Turab, Mustafa Asghar Awan, Linta Masroor, Khadija Amir, Mahnoor Khan, Afrose Liaquat

**Affiliations:** 1 College of Medicine, Shifa Tameer-E-Millat University Shifa College of Medicine, Islamabad, PAK; 2 Biochemistry, Shifa Tameer-E-Millat University Shifa College of Medicine, Islamabad, PAK

**Keywords:** gastroesophageal reflux disease (gerd), heartburn, lifestyles diseases, medical school, medical students, psychological stress, regurgitation, stress scales, youth

## Abstract

Background: Gastroesophageal reflux disease (GERD) is a common complaint seen in medical clinics today, affecting countless people worldwide. Patients can present with a wide array of symptoms, ranging from classical complaints like heartburn and acid reflux to atypical cases like silent GERD. Recent advancements in diagnostic testing and imaging have aided in diagnosing and understanding GERD. Given the prevalence of GERD and stress within the confines of our society, it is pertinent to establish a correlation between these two, which this study aimed to investigate.

Methods: A cross-sectional study was conducted using the Gastroesophageal Reflux Disease Questionnaire (GERDQ), the Perceived Stress Scale (PSS), and the Student Stress Inventory (SSI), combined in a survey distributed to participants online. These included students from various healthcare institutes.

Results: A total of 439 responses were analyzed, of which 262 (59.7%) were female and 177 (40.3%) were male. Of them, 319 (72.7%) were day scholars, and 120 (27.3%) were students living in hostel accommodation. The mean age was 20.5 years. Female participants had a significantly higher prevalence of GERD (16.4%, n = 43) than male participants (9.6%, n = 17) (p < 0.05). Similar trends were seen in both stress scales, with the female group showing a significantly higher proportion of high-stress individuals (p < 0.05). Program of study, age, accommodation, year of study, hours of sleep, and hours of study had no significant impact on the presence of GERD. However, when the same variables were analyzed against levels of stress, the mean hours of study and mean hours of sleep were significant in the SSI (p < 0.05). The high-stress group in the SSI had a significantly higher mean sleep duration (7.93 hours) and mean study duration (5.77 hours) than both the moderate-stress and low-stress groups.

Conclusion: Results of this study show that increased stress has a positive correlation with the prevalence of symptoms of GERD. Steps should be taken to reduce stress among medical students to alleviate such symptoms.

## Introduction

Gastroesophageal reflux disease (GERD) is broadly defined as a condition characterized by a variety of symptoms, ranging from heartburn and regurgitation to bloating, dyspepsia, and abdominal discomfort [1]. The lack of a formal definition, however, makes it difficult to diagnose GERD with high accuracy. It has been reported that the global prevalence of GERD is 14% and varies by region. The highest region-based prevalence, incidence, and years lived with disease (YLDs) have been observed in the Middle East, North Africa, South Asia, and East Asia, whereas the lowest prevalence was reported in the Caribbean and Oceania [2]. In Pakistan alone, a few studies have reported the prevalence of GERD to range between 10% and 64% [3-5].

Among other causes of GERD, psychological stress has been found to have a strong association with GERD symptoms [6]. Medical school is recognized as a stressful environment that negatively affects students' psychological well-being [7]. Studies conducted in different countries have found the prevalence of psychological stress to be 51.9% in Nigeria [8], 88.9% in Egypt [9], 56% in Malaysia [10], and 71.9% in Saudi Arabia [11]. A study conducted in Karachi, Pakistan, reported anxiety and depression levels of approximately 70% among medical students, while a more recent study found stress in this subset of the population to be 54.6% [12].

This study aims to evaluate the prevalence of GERD and stress among Pakistani medical students. It also seeks to analyze their association with sociodemographic factors and provide insight into strategies for managing stress better to aid in the prevention and treatment of GERD.

Given the significant prevalence of both GERD and psychological stress within the population, it has become increasingly important to investigate and establish whether an association exists between these two conditions. Understanding this potential relationship could lead to more effective management strategies, where targeted stress reduction interventions may help alleviate GERD symptoms and improve overall patient outcomes.

## Materials and methods

An analytical cross-sectional study, approved by the Institutional Review Board (IRB #0253-22) at Shifa International Hospital, Islamabad, was carried out from February 2023 to September 2023 in the twin cities of Rawalpindi and Islamabad. A total of 463 students from various medical and dental universities across all academic years participated in the study. Using OpenEpi and an estimated GERD prevalence of 60%, the sample size was calculated to be 273, with 95% confidence intervals. All medical and dental universities were included in this study, with the minimum participant age set at 18 years. Forms submitted by students from allied health sciences (excluding MBBS and BDS) and those that were incompletely filled were discarded. Non-probability convenience sampling was used.

Questionnaires

The questionnaire used consisted of a checklist of demographic information, including gender, age, program, year of study, living status, hours of study, and hours of sleep. Alongside this, previously validated questionnaires, such as GERD Questionnaire (GERDQ), Perceived Stress Scale (PSS), and Student Stress Inventory (SSI), were used to evaluate the presence of GERD and stress [13-15]. The combined questionnaire was shared online using Google Forms. Responses that were filled out incompletely were discarded. Those submitted by non-medical students were also removed and deleted, as they were not required for the study.

The GERDQ is a validated tool used to determine the risk of GERD. It comprises six items, including two inversely scored ones, all rated on a Likert scale from 0 to 3, depending on the frequency in the previous week. After the total is calculated, a GERDQ score of 8 is considered positive for the diagnosis of GERD, with 65% sensitivity and 71% specificity [13].

The PSS is a validated tool consisting of 10 items, rated on a Likert scale from 0 to 4. A total of four items require their values to be inverted before being added to the final score. The scores are divided into three ranges: 0-13 for low stress, 14-26 for moderate stress, and 27-40 for high perceived stress [14].

The SSI is a 40-item self-reported survey that assesses student stress. The scale comprises four subscales: physical, interpersonal relationships, academic, and environmental. Each subscale consists of 10 items, with responses ranging from 1 to 4. The total response scores range from 40 to 160, with higher scores indicating greater stress [15]. For comparison, the scores were grouped into three ranges: 40-80 for low stress, 81-120 for moderate stress, and 121-160 for high stress.

Analysis

The data were then organized and analyzed using IBM SPSS Statistics for Windows, Version 26 (Released 2019; IBM Corp., Armonk, New York). For categorical variables, the chi-square test was applied, and for continuous variables, the t-test was used. The chi-square test was used to cross-examine the prevalence of GERD and high perceived stress. For the other variables, the chi-square test and independent samples t-test were used to identify any significant association between these variables and GERD or stress. A p-value of less than 0.05 was considered statistically significant.

## Results

**Figure 1:**
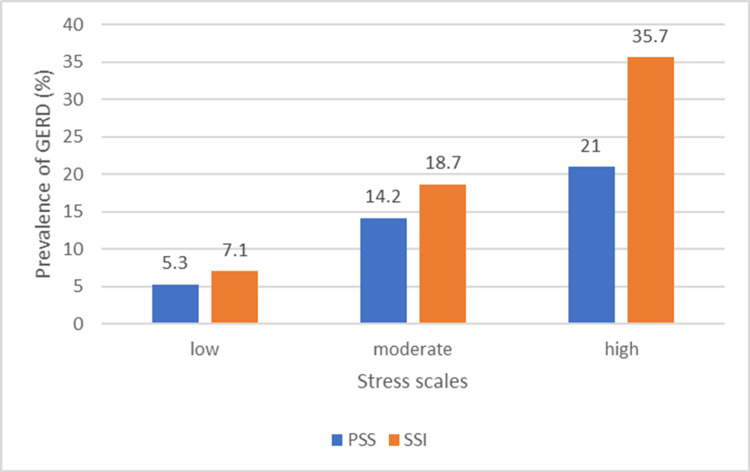
Prevalence of GERD across PSS and SSI GERD: gastroesophageal reflux disease, PSS: Perceived Stress Scale, SSI: Student Stress Inventory.

A total of 463 participants filled out the questionnaires, of which 24 were discarded as they were incomplete. The final dataset included 439 participants, of whom 177 (40.3%) were male and 262 (59.7%) were female. Among them, 319 (72.7%) were day scholars, while 120 (27.3%) were students living in hostel accommodation. The majority of the sample (358 participants, 81.5%) was enrolled in MBBS programs, while the rest were dispersed across other programs. The mean age was 20.5 years. Figure 1 summarizes the increasingly linear trend between GERD and stress across both stress scales.

Gastroesophageal Reflux Disease Questionnaire

Among the 439 participants, the prevalence of GERD was 13.6% (n = 60). Students with moderate or high stress levels were 364 (82.9%) on the PSS and 228 (51.9%) on the SSI. Table 1 summarizes all demographic profiles as risk factors for GERD. Among these, the most significant variable was stress (p < 0.05).

**Table 1:** Prevalence of GERD alongside different variables The data are presented as numbers and percentages. Chi-square and independent sample t-tests were used. A p-value of <0.05 was considered significant. GERD: gastroesophageal reflux disease, SSI: Student Stress Inventory, PSS: Perceived Stress Scale, MBBS: Bachelor of Medicine and Bachelor of Surgery.

Variables	Presence of GERD (N=60)	Absence of GERD (N=379)	p-value
SSI			0.000
Low stress (n=211)	15 (25%)	196 (51.7%)	
Moderate stress (n=214)	40 (66.7%)	174 (45.9%)	
High stress (n=14)	5 (8.3%)	9 (2.4%)	
PSS			0.026
Low stress (n=75)	4 (6.7%)	71 (18.7%)	
Moderate stress (n=302)	43 (71.7%)	259 (68.3%)	
High stress (n=62)	13 (21.7%)	49 (12.9%)	
Female gender (n=262)	43 (71.7%)	219 (57.8%)	0.041
Male gender (n=177)	17 (28.3%)	160 (42.2%)	
MBBS-enrolled (n=358)	45 (75.0%)	313 (82.6%)	0.409
Hostel residents (n=120)	21 (35.0%)	99 (26.1%)	0.152
Year of study			0.441
First year (n=63)	4 (6.7%)	59 (15.6%)	
Second year (n=113)	14 (23.3%)	99 (26.1%)	
Third year (n=135)	22 (36.7%)	113 (29.8%)	
Fourth year (n=67)	9 (15.0%)	58 (15.3%)	
Fifth year (n=28)	5 (8.3%)	23 (6.1%)	
Outgoing fifth year (n=33)	6 (10.0%)	27 (7.1%)	
Hours of sleep (mean)	6.51	6.71	0.227
Hours of study (mean)	3.44	3.13	0.662
Mean age (years)	20.78	20.45	0.642

Perceived Stress Scale

On the PSS, among students in the low-stress group, only 4 (5.3%) had GERD. In the moderate-stress group, 43 (14.2%) had GERD. Similarly, in the high-stress group, 13 (21.0%) had GERD. The chi-square value was 7.299 (X² = 7.299), which was greater than the critical value of 5.991 (X² > 5.991), leading to rejection of the null hypothesis. This indicates an increasing linear relationship between perceived stress levels and the presence of GERD.

Student Stress Inventory

On the SSI scale, among students in the low-stress group, only 15 (7.1%) had GERD. In the moderate-stress group, 40 (18.7%) had GERD, and in the high-stress group, 5 (35.7%) had GERD. The chi-square value was 18.037 (X² = 18.037), which was greater than the critical value of 5.991 (X² > 5.991), leading to rejection of the null hypothesis. This indicates an increasing linear relationship between student stress levels and the presence of GERD.

Female participants had a significantly higher prevalence of GERD (16.4%, n = 43) than male participants (9.6%, n = 17) (p < 0.05). Similar trends were observed in both stress scales, with females showing a much higher proportion of high-stress individuals (p < 0.05). Program of study, age, accommodation, year of study, hours of sleep, and hours of study had no significant impact on the presence of GERD. However, when the same variables were analyzed against levels of stress, mean hours of study and mean hours of sleep were significant in the SSI (p < 0.05). The high-stress group in the SSI had a significantly higher mean sleep duration (7.93 hours) and mean study duration (5.77 hours) than both the moderate-stress and low-stress groups (Table 2). None of the demographic factors were significantly related to stress in the PSS scale (p > 0.05) (Table 3).

**Table 2:** Prevalence of stress among different variables (SSI) The data are presented as numbers and percentages. Chi-square and independent sample t-tests were used. A p-value of <0.05 was considered significant. SSI: Student Stress Inventory.

Variables	SSI low stress (N=211)	SSI moderate stress (N=214)	SSI high stress (N=14)	p-value
Year of study				0.336
First year (n=63)	32 (15.2%)	30 (14.0%)	1 (7.1%)	
Second year (n=113)	55 (26.1%)	54 (25.2%)	4 (28.6%)	
Third year (n=135)	55 (26.1%)	76 (35.5%)	4 (28.6%)	
Fourth year (n=67)	39 (18.5%)	25 (11.7%)	3 (21.4%)	
Fifth year (n=28)	11 (5.2%)	15 (7.0%)	2 (14.3%)	
Outgoing fifth year (n=33)	19 (9.0%)	14 (6.5%)	0 (0%)	
Gender				0.000
Male (n=177)	106 (50.2%)	67 (31.3%)	4 (28.6%)	
Female (n=262)	105 (49.8%)	147 (68.7%)	10 (71.4%)	
Hostel residents (n=120)	62 (29.4%)	55 (25.7%)	3 (21.4%)	0.613
MBBS-enrolled (n=358)	174 (82.5%)	172 (80.4%)	10 (71.4%)	0.470
Hours of sleep (mean)	6.75	6.54	7.93	0.003
Hours of study (mean)	3.08	3.11	5.43	0.001
Mean age (years)	20.51	20.50	20.29	0.863

**Table 3:** Prevalence of stress among different variables (PSS) The data are presented as numbers and percentages. Chi-square and independent sample t-tests were used. A p-value of <0.05 was considered significant. PSS: Perceived Stress Scale.

Variables	PSS low stress (N=75)	PSS moderate stress (N=302)	PSS high stress (N=62)	p-value
Year of study				0.716
First year (n=63)	10 (13.3%)	46 (15.2%)	7 (11.3%)	
Second year (n=113)	23 (30.7%)	73 (24.2%)	17 (24.7%)	
Third year (n=135)	23 (30.7%)	92 (30.5%)	20 (32.3%)	
Fourth year (n=67)	13 (17.3%)	45 (14.9%)	9 (14.5%)	
Fifth year (n=28)	1 (1.3%)	21 (7.0%)	6 (9.7%)	
Outgoing fifth year (n=33)	5 (6.7%)	25 (8.3%)	3 (4.8%)	
Gender				0.000
Male (n=177)	49 (65.3%)	112(37.1%)	16(25.8%)	
Female (n=262)	26 (34.7%)	190 (62.9%)	46 (74.2%)	
Hostel residents (n=120)	18 (24.0%)	84 (27.8%)	18 (29.0%)	0.762
MBBS-enrolled (n=358)	62 (82.7%)	246 (81.5%)	50 (80.6%)	0.294
Hours of sleep (mean)	6.89	6.57	7.00	0.063
Hours of study (mean)	2.75	3.23	3.40	0.186
Mean age (years)	20.68	20.43	20.61	0.346

## Discussion

Our cross-sectional study aimed to find the association between psychological stress and the prevalence of GERD in medical and dental students in Islamabad and Rawalpindi. Psychological stress is said to occur when an individual feels that environmental demands exceed their adaptive capacity [16]. A population-based cross-sectional case-control study conducted in Nord-Trøndelag, Norway, using data from two health surveys, showed a positive association between GERD symptoms and certain psychosocial factors (high job demand, low job control, and job strain, etc.), demonstrating that stress, regardless of its cause, is directly related to the presence of GERD symptoms [17].

As reported by many studies, perceived stress is experienced at moderate to severe levels in medical students due to academic and psychosocial factors [18]. These academic factors include tests and exams, which, despite being a primary source of stress for many students, are essential in their training and evaluation. Psychosocial factors contributing to stress in medical students include, but are not limited to, parental expectations, frequency of examinations, sleeping difficulties, the vastness of the academic curriculum, loneliness, and concerns about the future [18].

Multiple mechanisms have been proposed to explain how stress could exacerbate GERD symptoms. Increased psychological stress may lead to certain lifestyle changes, including smoking, alcohol consumption, comfort food consumption, physical inactivity, and insomnia, which in turn could worsen GERD symptoms [19]. Alcohol and tobacco smoking have been suggested to decrease lower esophageal sphincter tone, leading to GERD symptoms [19]. Another proposed explanation for why individuals with stress experience greater GERD symptom severity is heightened sensitivity to stimuli, causing low-intensity esophageal stimuli to be perceived as painful reflux symptoms. It is also believed that stress increases the permeability of the esophageal mucosa, contributing to reflux esophagitis [19].

Our study used two stress scales, the Perceived Stress Scale (PSS) and the Student Stress Inventory (SSI), to assess stress levels in medical students. We found that a high number of students suffered from moderate to high levels of stress. Another study conducted among medical students reported similar results, with considerable symptoms of stress observed in undergraduate medical students [20]. Multiple reasons for stress in medical and dental schools have already been identified. However, this study found no statistically significant relationship between stress and the number of hours slept. This could be explained by several factors. Firstly, our research focused only on the quantity of sleep (in hours) and did not assess sleep quality. Medical students might experience disrupted sleep patterns or poor sleep quality due to stress, which could affect their overall well-being more than the total hours slept. Similarly, some students may cope with stress in ways that do not impact their sleep duration, while others show more pronounced effects. Variability in stress responses could obscure a clear relationship.

Using GERDQ and a cutoff value of 8 to classify participants as having GERD, we found the prevalence of GERD in our sample to be 13.6% (n = 60). This was very close to the prevalence found in undergraduate medical students in Baghdad (13.3%) [21]. However, other studies reported a higher prevalence, such as that by Sharma et al. [22], which reported a prevalence of 25%, and Memon et al. [23], which found it to be 70.4%. This discrepancy could be explained by several factors that were not assessed in our study. Adoption of a healthier lifestyle and dietary choices may have contributed to a lower prevalence of GERD in our sample. Similarly, engagement in regular physical activity could have played a role. It is also possible that greater awareness of GERD and easier access to over-the-counter medications to alleviate symptoms resulted in a lower prevalence than expected.

In this study, we found the prevalence of moderate to severe stress to be 82.9% (n = 364) on the PSS and 51.9% (n = 228) on the SSI. Another study conducted in Sialkot, Pakistan, reported similar findings, with moderate to extremely severe stress having a prevalence of 51.4% [24]. The causes of high stress levels could include academic pressure, with extensive studying, exams, and high expectations. Heavy workloads and the need for better time management could also be contributing factors. Additionally, the high cost of medical education and uncertainty about the future may explain the high stress levels observed in our study.

Among participants classified as having GERD, we found a positive relationship between perceived stress levels and the presence of GERD using both the PSS and SSI scales. As stress levels increased in intensity, so did the prevalence of GERD in each category. Jang et al. [25] identified two main reasons to explain the relationship between psychosocial factors and GERD. First, GERD could cause secondary depression and anxiety, increasing sensitivity to GERD symptoms. Second, genetic susceptibility to GERD is much higher in individuals who are more vulnerable to psychosocial stressors. Therefore, multiple factors contribute to the relationship between psychosocial stress and GERD, making it difficult to define through a single model [26].

Our study found a higher prevalence of GERD in females than males, with a statistically significant difference. Similar findings were reported by Manterola et al. in their cross-sectional study in southern Chile, where 64.3% of females had GERD compared to 26.9% of males [27]. However, Halawani et al. did not find any statistically significant correlation between gender and GERD [28]. Similar trends were also observed between gender and perceived stress, with female students reporting higher levels of stress on both stress scales. Higher stress levels among females than males have also been reported by Thawabein and Quaisy [29].

In this study, we did not identify any correlation between age and the presence of GERD. Baklola et al. also found similar results, reporting no association between GERD and age [30]. Additionally, no correlation was found between program of study, accommodation (hostel residence versus home accommodation), year of study, hours of study, or hours of sleep and the presence of GERD.

Our results did show a positive relationship between stress and the prevalence of GERD. We also explored associations with other factors, including demographic details, hours of sleep, living status, and hours of study, to provide a more comprehensive understanding of their relationship with stress and/or GERD. Moreover, we used two validated questionnaires to assess stress levels in our sample population, making this one of the few studies to cross-validate findings using multiple research tools.

This cross-sectional study was able to determine an association between stress and GERD symptoms; however, it was unable to definitively conclude that stress and GERD were causative of one another. The data collected from the questionnaires was based on self-reported answers, which, without quantitative medical diagnosis, can be subject to bias due to possible under- or over-reporting of symptoms by participants. While the sample size used for this study was calculated to attain appropriate results, this number cannot be generalized to the entire medical student population, since it only focuses on a specific region (Islamabad and Rawalpindi) and does not take into consideration other demographic factors at play over a wider population and region. As the survey was shared via Google Forms, this may introduce selection bias due to the limitation of students without reliable internet access. Additionally, this study did not take into account the quality of sleep or quality of study in students. While the GERDQ questionnaire has a high predictive value, it does not have much diagnostic value when used without a detailed history and quantitative medical testing. In addition, this study does not consider potential confounding variables, such as diet, lifestyle, genetic predispositions, or other comorbidities, that could influence the development of GERD.

This study demonstrates a positive relationship between perceived stress and GERD symptoms in medical students, suggesting that stress reduction strategies could help alleviate GERD symptoms. Reduction of stress could be achieved with interventions such as lifestyle modifications and medications. There was no statistically significant association with other factors, including demographic details, hours of sleep, living status, and hours of study.

From the limitations of this study, we recommend that future research further investigate this relationship using clinically diagnosed GERD cases, rather than relying on questionnaires to categorize potential cases, and exploring their levels of stress in comparison to controls not suffering from clinically diagnosed GERD.

## Conclusions

This study highlights a significant positive correlation between perceived stress and the prevalence of GERD symptoms among medical students. The findings suggest that individuals experiencing higher stress levels are more likely to report symptoms of GERD. Steps should be taken to reduce stress among medical students to alleviate such symptoms.
